# Study of distortion-product otoacoustic emissions during hypothermia in humans

**DOI:** 10.1016/S1808-8694(15)30575-9

**Published:** 2015-10-19

**Authors:** Andrei Borin, Oswaldo Laércio Mendonça Cruz

**Affiliations:** 1 MSc, Graduate Student - PhD.; 2 Professor at USP/SP, Associate Professor of otorhinolaryngology at UNIFESP/EPM. Department of Otorhinolaryngology and Head and Neck Surgery - Paulista Medical School - UNIFESP - São Paulo SP Brazil

**Keywords:** extra-corporeal circulation, hair-cells, cochlea, otoacoustic emissions, hypothermia

## Abstract

**Aim:**

To evaluate the function of cochlear outer hair-cells under the influence of extra-corporeal circulation and moderate hypothermia during cardiac surgery.

**Study Design:**

Prospective clinical study.

**Methods:**

Distortion-product otoacoustic emissions (DPOAE) were registered before surgery, immediately after general anesthesia induction, during extra-corporeal circulation with moderate hypothermia and after the surgical procedure. Results: Comparison of response-amplitudes before and after surgery and before and after general anesthesia initiation did not demonstrate statistical difference. Comparison of amplitudes before and after extra-corporeal circulation with moderate hypothermia demonstrated a statistically significant decrease in responses amplitudes during hypothermia.

**Conclusions:**

The amplitudes of DPOAE decreased during moderate hypothermia induced during extra-corporeal circulation.

## INTRODUCTION

Otology is faced with a new challenge this millennium: the study of the inner ear. This portion of the auditory apparatus has posed the largest obstacles to reaching accurate diagnosis and effective treatment. The outer hair cells (OHC) in the cochlea have been extensively analyzed in areas such as cochlear microfonism, sound amplification and frequency discrimination^1^. One of the methods to study the OHC is recording otoacoustic emissions (OAE)^1^, ^2^.

Studies have reported the marked susceptibility of the OHC to hypovolemia and/or ischemia^3^. In order to further investigate the matter, we decided to look into distortion product otoacoustic emissions (DPOAE) during heart surgery with extracorporeal circulation (ECC) and hypothermia, an event where the risk of peripheral organ ischemia is high in spite of technological progress, as it changes the pulsating blood flow driven by the heart to a continuum produced by the machine. Additionally, during such procedures patients are usually submitted to moderate (28-29ºC) or deep (18ºC) hypothermia. This is the setting in which the study of ECC in situations of hypoperfusion and hypothermia becomes invaluable to better understand how these cells function under stressful circumstances.

The risk of sensorineural hearing loss after surgical procedures including ECC and hypothermia is estimated at 0.14% in the literature, a rate six times greater that the risk incurred in by the population in general^4^, ^5^. Could DPOAE, as it mimics OHC, be utilized to monitor such risk? It has been established in the literature that hypothermia impacts cochlear potential, spontaneous OAE and evoked transients, both in animal and human models^6^, ^7^, ^8^, ^9^, ^10^, ^11^, ^12^. However, there are no reports on altered DPOAE test results during hypothermia in humans. The purpose of this study is to assess the behavior of the OHC during heart surgery where ECC and moderate hypothermia are used through DPOAE.

## MATERIALS AND METHODS

This study was approved by the Research Ethics Committee at our institution under permit 0592/02.

The protocol described below was instituted to study the behavior of DPOAEs in heart surgery patients submitted to moderate hypothermia. Before surgery, all patients were interviewed and asked about age, gender, place of birth, occupation, medical and audiological history; they also underwent a thorough otorhinolaryngological examination. In order to qualify for the trial, patients had to have normal otorhinolaryngological findings, no auditory complaints, no previous history of acoustic trauma and/or exposure to noise, and be able to comprehend (either the patient of his/her legal representative) and sign an Informed Consent term.

Within four months, eighteen patients that would be operated on by the same team of heart surgeons and anesthesiologists were interviewed. One of these patients refused to participate in the trial, another had a perforated eardrum and was thus excluded, and two had their surgeries called off for clinical reasons. In all, fourteen patients were included in the trial (28 ears), ten males and four females. Their ages ranged between 26 and 62 years of age and averaged at 47.86 years (STD = 14.5 years).

Three consecutive DPOAE measurements were done on each ear by the patients’ beds, in a silent however not acoustically insulated environment; this data series was named ’PRE’. During surgery DPOAE tests were done in two stages: after anesthesia - three consecutive measurements on each ear, data series ’ANESTHESIA’; and after ECC was in place with moderate hypothermia - three consecutive measurements on each ear, data series ’HYPOTHERMIA’. Between three to five days after surgery, as the patients had been sent back to their beds, three additional consecutive measurements were done on each ear under the ’POST’ data series.

All surgical procedures were carried out in the same operating room by the same team of heart surgeons. Anesthesia was administered intravenously, without nitrous oxide. ECC was provided by a heart-lung Biomedica BEC 2000 System® machine, a continuous flow system with roller head pumps, venous and arterial tubing, and membrane oxygenator. The patients were anticoagulated and their bodily temperatures monitored with a pharyngeal thermometer.

For the DPOAE tests a portable Grason-Stadler Inc.® device with GSI-60 software was used, and the Darmuth Hitchcock Medical Center protocol by Musiek e Baran em 1997^13^ adopted, with two tones L1 = L2 = 70 dB of sound pressure level (SPL) and f2/f1 = 1.22. The tests were analyzed using a ’DPgram’ by amplitude of response (A) of the distortion product (DP) in relation to background noise (BN). The measurement [A = DP - BN] in dB of SPL was obtained for frequency 2f1 - f2 in a total of 11 points corresponding to 531, 687, 843, 1093, 1375, 1750, 2187, 2781, 3500, 4375 and 5500 Hz (called F1 to F11 respectively) related to the geometric average of f1 and f2 used to build the ’DPgram’. Occurrences of DPOAE were considered positive when ’A’ was equal to or greater than 3 dB SPL. Data series PRE, ANESTHESIA, HYPOTHERMIA, and POST were analyzed for each ear and each of the eleven points of the ’PDgram’ listed above (F1 to F11).

On each of the four series, three tests were done for each ear, but to be deemed valid for analysis purposes the following set of criteria was applied:

§ If two or three records were ’rejected’ by the device, the data was deemed ’invalid’ for analysis.
§ If at least two records were ’accepted’ by the device:
  • Two or three values of A ≥ 3 dB SPL - DPOAE present and the averages were calculated;
  • Two or three values of A < 3 dB SPL - DPOAE absent.

With that, one average value was defined for each of the eleven points of the ’DPgram’ (F1 to F11) from the PRE, ANESTHESIA, HYPOTHERMIA, and POST data series. For descriptive statistics, all continuous variables were described in terms of average, standard deviation, variation amplitude, maximum and minimum values, median, upper and lower quartiles, skewness, kurtosis, and the Kolmogorov-Smirnov test.

In order to analyze the significance of the differences found on each of the series and on each of the points of the ’DPgram’ (F1 to F11), the averages for both ears of the patients were observed. If only one of the ears provided for a valid record, the data from such ear was considered for analysis. When only two ranges of values were available, the ’Paired t-Test’ was used. When more than two ranges of values were observed, the ’one-way ANOVA’ and ’all pairwise multiple comparison procedures - Dunnett´s method’ were used to compare the PRE data series to all others (PRE x POST, PRE x ANESTHESIA, PRE x HYPOTHERMIA).

The possibility of performing parametric tests was assessed through the Kolmogorov-Smirnov Normality Test or variance equivalence test. When parametric tests could not be applied, logarithmic (Log10 e Ln) and squaring transformations were tried, and then again normality was tested. When impossibility prevailed, the comparison was done through Friedman's Repeated Measures Analysis of Variance on Ranks. The cutoff point for significance was 0.05. Statistical and graphic calculations were done on the following registered software: STATISTICA 4.5® (Statsoft, Inc. 1993) and SIGMA STAT 1.0® (Jandel Corporation, 1993, 956245).

## RESULTS

**Chart 1:**
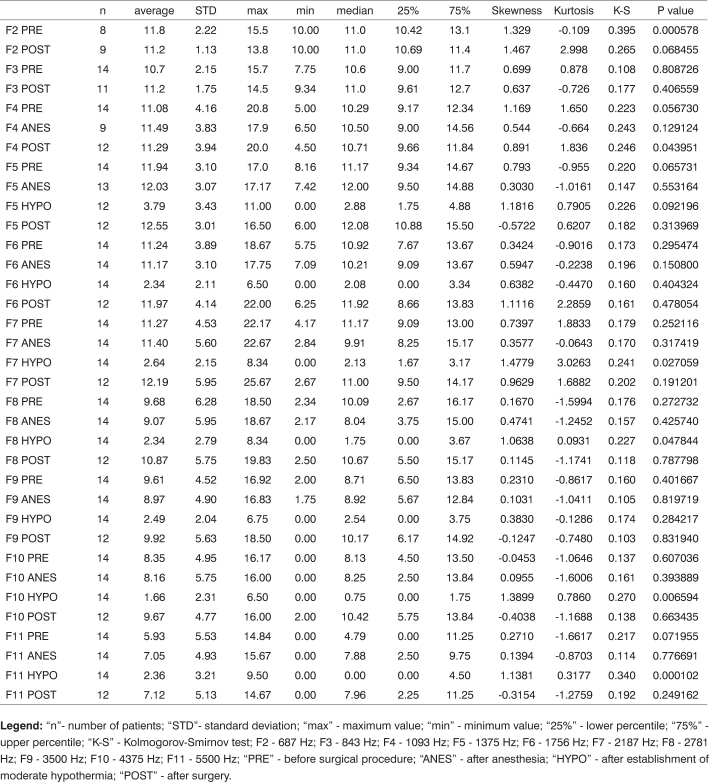
Descriptive statistics for each point in the ’DPgram’ (F2 to F11) and each data series (PRE, ANESTHESIA, HYPOTHERMIA, and POST).

**Legend:** “n”- number of patients; “STD”- standard deviation; “max” - maximum value; “min” - minimum value; “25%” - lower percentile; “75%” - upper percentile; “K-S” - Kolmogorov-Smirnov test; F2 - 687 Hz; F3 - 843 Hz; F4 - 1093 Hz; F5 - 1375 Hz; F6 - 1756 Hz; F7 - 2187 Hz; F8 - 2781 Hz; F9 - 3500 Hz; F10 - 4375 Hz; F11 - 5500 Hz; “PRE” - before surgical procedure; “ANES” - after anesthesia; “HYPO” - after establishment of moderate hypothermia; “POST” - after surgery.

**Chart 2:**
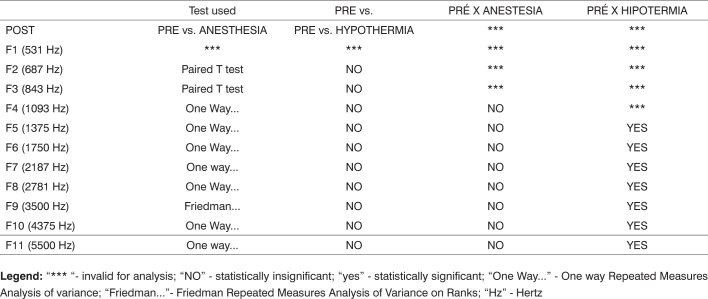
Statistical analysis summary for results of each point in the ’DPgram’ showing statistically significant differences (p<0.05).

**Legend:** “*** “- invalid for analysis; “NO” - statistically insignificant; “yes” - statistically significant; “One Way...” - One way Repeated Measures Analysis of variance; “Friedman...”- Friedman Repeated Measures Analysis of Variance on Ranks; “Hz” - Hertz

**Graph 1:**
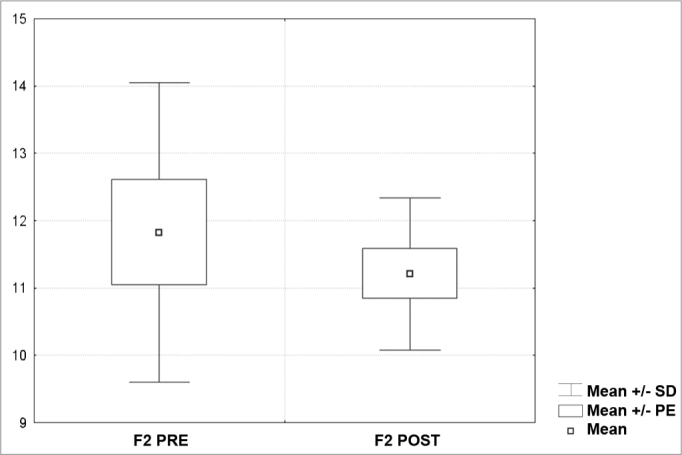
“Boxplot” for F2 (687 Hz) - values in dB SPL

**Graph 2:**
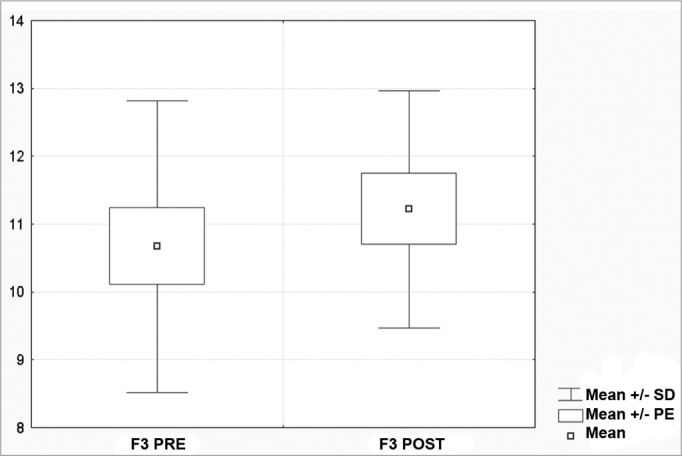
“Boxplot “ for F3 (843 Hz) - values in dB SPL

**Graph 3:**
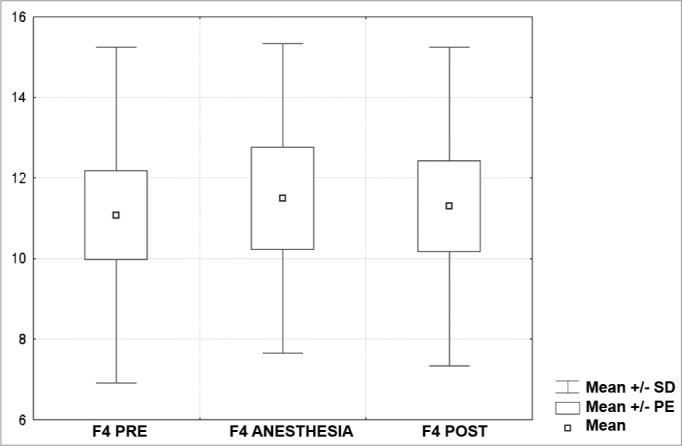
“Boxplot “ for F4 (1093 Hz) - values in dB SPL

**Graph 4:**
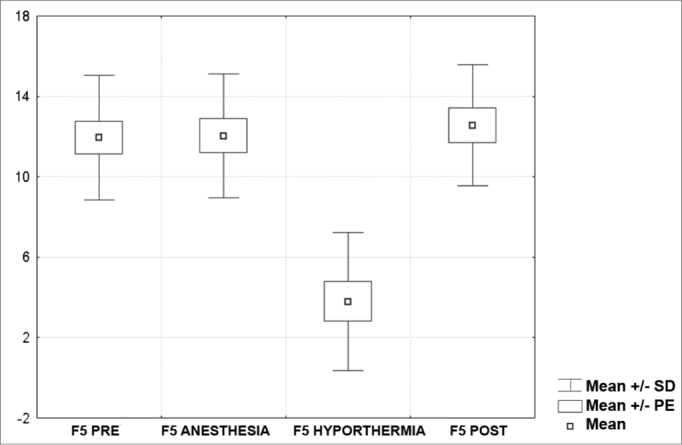
“Boxplot “ for F5 (1375 Hz) - values in dB SPL

**Graph 5:**
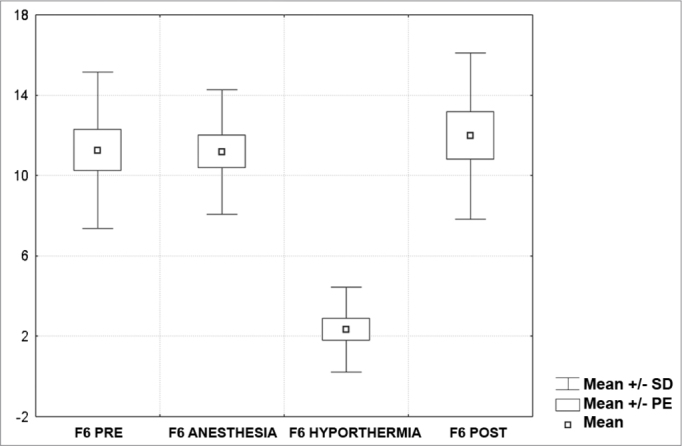
“Boxplot “ for F6 (1750 Hz) - values in dB SPL

**Graph 6:**
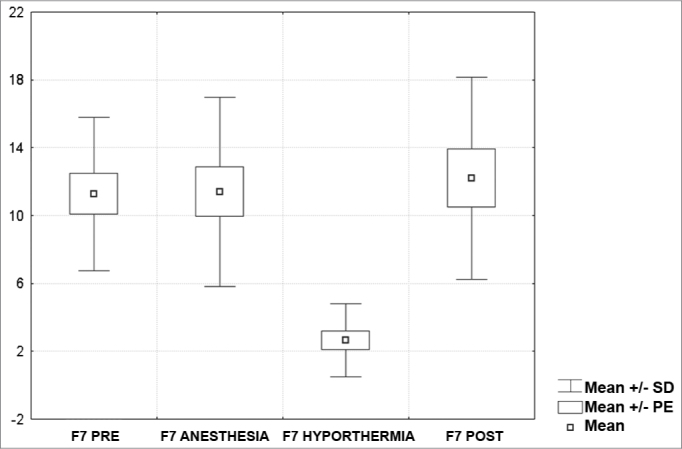
“Boxplot “ for F7 (2187 Hz) - values in dB SPL

**Graph 7:**
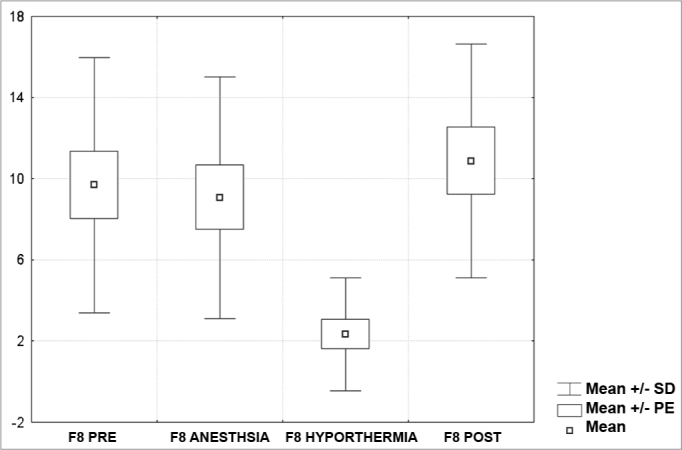
“Boxplot “ for F8 (2781 Hz) - values in dB SPL

**Graph 8:**
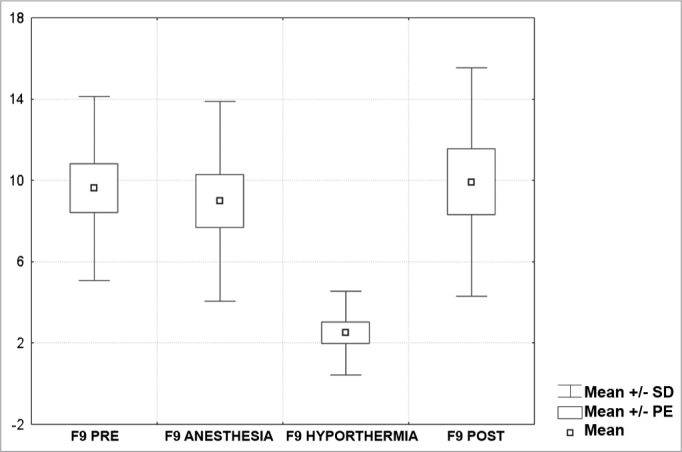
“Boxplot “ for F9 (3500 Hz) - values in dB SPL

**Graph 9:**
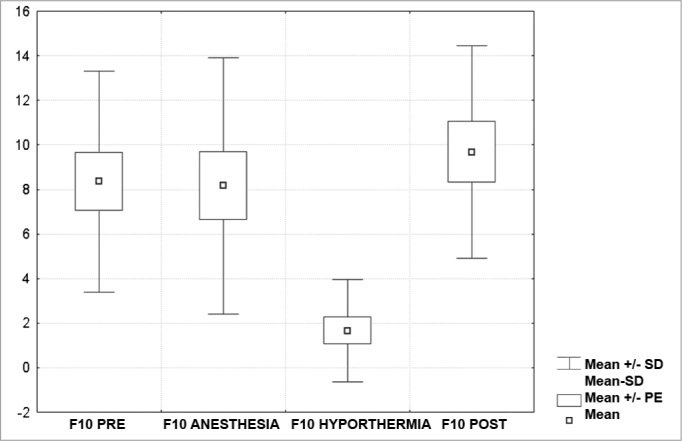
“Boxplot “ for F10 (4375 Hz) - values in dB SPL

**Graph 10:**
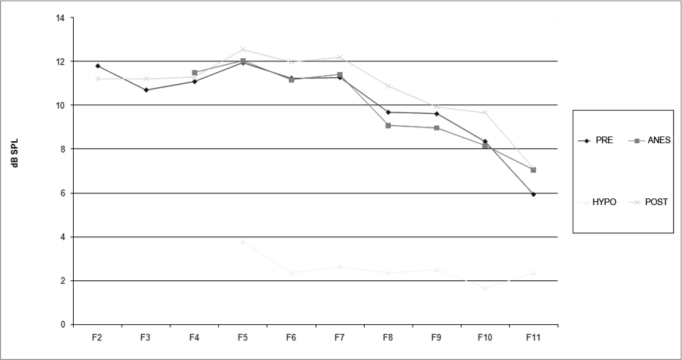
“Boxplot “ for F11 (5500 Hz) - values in dB SPL

**Graph 11:**
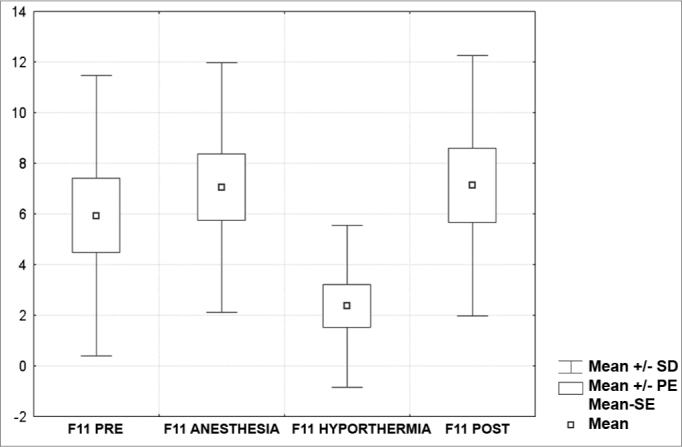
Comparison between DPOAE averages for each studied frequency. Values in dB SPL.

Chart 1 comprises the description of the DPOAE amplitudes for each point in the ’DPgram’ (F2 to F11) and each data series (PRE, ANESTHESIA, HYPOTHERMIA, and POST). F1 (531Hz) was not included, as no valid data was obtained for analysis. Data points also considered invalid for analysis: F2 ANESTHESIA, F2 HYPOTHERMIA, F3 ANESTHESIA, F3 HYPOTHERMIA and F4 HYPOTHERMIA. A summary of the comparative analysis can be seen in Chart 2, including all statistically significant data. For each point in the ’DPgram’ a ’box plot’ was presented (with values expressed in dB SPL) (Graph 1, Graph 2, Graph 3, Graph 4, Graph 5, Graph 6, Graph 7, Graph 8, Graph 9, Graph 10). By comparing data series PRE and POST, no differences were found for any of the points. Such absence of significance was also observed when comparing the PRE and ANESTHESIA data series. On the other hand, when comparing PRE and HYPOTHERMIA, reduced DPOAE amplitudes were found (p<0.05) for points F5 to F11, for which valid data points were available. Graph 11 shows the behavior of the averages on each of the frequencies for the PRE, ANESTHESIA, HYPOTHERMIA, and POST data series.

## DISCUSSION

OAEs, described by Kemp in 1978^14^, arise apparently in the OHC^2^, ^15^, ^16^ and possibly represent the rapid contractions of this cell group. They are acoustic phenomena of cochlear nature, which reverberate through the ossicles in the middle ear and are transmitted to the ear canal, where they can be captured through a microphone. This spurt of energy in the form of sound may occur spontaneously or in response to external auditory simulation. Although these cells contain actin and myosin filaments similar to those found in muscle cells, it is believed that the rapid contractions that produce OAEs do not stem from this mechanism. They occur in the absence of calcium, a necessary ion for actin and myosin to couple together and act as a contractile unit. Various explanations for the likely contraction mechanism in place have been discussed, such as the participation of the lateral cisterns^17^, cortical cytoskeleton^15^, and cell membrane proteins^18^.

Also in 1978, Kemp^14^ defined the cochlea as a non-linear sound amplifier that intermodulates the pure tones used in the DPOAE tests and responds through a series of sounds in different frequencies, the most prevalent being the one corresponding to 2f1-f2, which was chosen to record the phenomenon. The precise site in the cochlea where DPOAEs are generated remains controversial. Lonsbury-Martin et al.^19^, Probst and Harris^20^, Beattie and Jones^21^ and Doyle et al.^22^ believe they originate in the cochlear region that corresponds to f2 or in the region that corresponds to the geometric average of f1 and f2. However, at least in animal models, other candidate sites have been discussed^23^. This controversy, however, does not reduce its application in the study of the effect produced by ototoxic drugs^24^, ^25^, noise induced auditory disorders^26^, inner ear diseases^1^, and other situations in which cochlear injury may occur^27^.

No acceptable DPOAE records were obtained at lower frequencies due to excessive background noise, a fact also reported and studied by other authors. Delgado et al.^28^ published a paper on the development of software to minimize the impact background noise when DPOAE tests are run in hospital settings without the ideal acoustic treatment. Internal (breathing, mastication, heartbeats) and external (environment) noise may contaminate the DPOAE test, mainly in lower frequencies. This was clearly observed in the ANESTHESIA and HYPOTHERMIA series, as the data was collected in the operating room where low frequencies simply could not be properly recorded. A similar situation was described by LeBourgeouis III et al.^29^.

Heart surgery with ECC modifies circulatory physiological patterns and exposes various organs to stress. The central nervous system (CNS) is the entity with highest exposure to ischemic injury in this context, with risk assessed between 2% and 61%^5^, ^30^, ^31^, ^32^, ^33^. Possible CNS injuries during surgery include: relatively low effectiveness of blood filters; occlusion of the descending aorta; microemboli formed from ruptured atherosclerotic plaques or coming from dilated heart chambers; air entering the system; fatty embolism; coagulation disorders; hypotension; and non-pulsing flow.

Our patients had increased risk of cochlear damage due to advanced age^34^ and associated diseases (high blood pressure, diabetes, and atherosclerosis). Ness et al.^4^, in a large cohort of heart surgery patients, detected pre-op auditory disorders in 77% of them. When looking at papers on post-ECC hearing loss^5^, ^35^, similar pathophysiologic mechanisms associated with CNS injury can be found, the main being inner ear hypoperfusion and ischemia. However, there are no pathology findings to support such assumptions. Others such as Ness et al.^4^ have attempted to explain post-ECC cochlear injury more as a consequence of ototoxic drug use than the surgical procedure itself. Meri et al.^36^ related post-ECC pulmonary complications to activation of the complement system, another mechanism that may occur at a cochlear level. Cochlear injury consequent to ECC has not been clearly defined. Among our patients we could not find subjective (complaints) or objective (PRE vs. POST) evidences of cochlear injury during ECC, because of two possible reasons. First, the DPOAE test may not be sensitive enough to capture it. Second, in our group of 14 patients there was no case of cochlear injury, as its prevalence is estimated at 0.14%^4^, ^5^. The second hypothesis seems more credible, as the DPOAE test is used routinely to monitor cochlear function in other circumstances, as mentioned previously.

In 1979, Anderson e Kemp^37^ demonstrated the occurrence of OAEs during general anesthesia in primates. DPOAE evaluation in agitated children is routinely done under sedation. In a study conducted in humans to assess the impact of general anesthesia upon the recording of evoked transient OAEs, Hauser et al.38 did not report pre or post anesthesia alterations. During anesthesia, they reported mild alterations mainly when nitrous oxide was being administered. Seifert et al.^12^, however, did not find general anesthesia to have affected the recording of evoked transient OAEs. In our study there was no detected impact of general anesthesia without nitrous oxide on the recording of DPOAEs when comparing the PRE vs. ANESTHESIA data series.

Coats^6^ mentioned in 1965 that cats under hypothermia had altered inner ear function as observed in the records of cochlear microfonics and action potentials. In 1985, Doyle and Fria^39^ reported altered brainstem audiometry results in monkeys under hypothermia, a finding also confirmed in humans by other authors^40^, ^41^. Other indicators of neurologic and cochlear activity such as EEG and P300 tests are also impacted by hypothermia, as CNS metabolism decreases along with axonal conduction velocity and changes to the synaptic cleft are observed^39^, ^40^, ^41^, ^42^. However, the effect of hypothermia on cochlear function can be better assessed by looking at OAEs.

In last century's last decade, several authors looked into the effects of hypothermia on the inner ear function of various animal models (amphibians, reptiles, birds, and mammalians)^7^, ^8^, ^9^, ^10^. We found studies on the impact hypothermia has on recording evoked transient OAE in humans^11^, but not DPOAEs. We believe that our study has clearly demonstrated the impact hypothermia has upon DPOAE based on the comparison between the PRE and HYPOTHERMIA data series, where a statistically significant amplitude reduction was identified for all points in the ’DPgram’ deemed valid (F5, F6, F7, F8, F9, F10, F11).

When facing these findings, three possible explanations may be considered:

1) changes in middle ear impedance during hypothermia hinder DPOAE acquisition in spite of their occurrence;
2) inhibited CNS activity during hypothermia leads to altered medial olivocochlear feedback and diminished OAE; and
3) changes in cochlear physiology during hypothermia and reduced OHC activity lad to reduced DPOAE.

Seifert et al.^12^ measured middle ear SPL during evoked transient OAE acquisition under hypothermia. Evoked transient OAEs disappeared at bodily temperatures of approximately 30ºC and middle ear SPL of -177 dPa. As the patients were warmed up, they reappeared at -300 dPa. Thus, it was concluded that middle ear SPL changes bear little significance upon study findings. Lonsbury-Martin et al.43 studied patients with normal hearing and detected a failure rate of 33% in recording DPOAEs. They resorted to tympanometry and stapedial reflex studies but did not succeed in correlating the failure rate to alterations in the middle ear. Le Bourgeois III et al.^29^, using animal models and looking at tympanic perforations of various sizes, were only able to find changes to DPOAE when perilymph fistulae were present. Therefore, although it may be said that changes in middle ear impedance during hypothermia could hinder DPOAE recording, we tend to agree with Seifert et al.^11^ and Kvolves et al.^10^ and conclude that OAE disappearance during hypothermia does not occur by the works of such mechanism.

When analyzing the second possible mechanism for reduced DPOAE during hypothermia, one must bear in mind that the olivocochlear system has marked impact upon OHC function^40^, ^41^. Such impact, however, inhibits OHC contraction as activity in the olivocochlear pathway is increased in the CNS^12^, ^16^. Thus, we do not believe that diminished CNS activity during hypothermia42 and reduced OHC inhibition by the medial olivocochlear system can account for DPOAE amplitude decrease. On the contrary, it seems more logical to believe that such fact would even facilitate amplitude increases, as is the case in brainstem audiometry tracings during mild hypothermia^40^, ^41^.

We believe that DPOAE amplitude decreases during hypothermia are related to cochlear alterations and decreased OHC activity. According to Seifert et al.^12^, the OHC contraction mechanism that leads to OAE does not energetically depend on adenosine triphosphate, but on the energy coming from the endocochlear potential. EP changes during hypothermia^44^ may be responsible for their decrease. Kvolves et al.^10^ have also pointed out to direct impact of hypothermia upon the OHC contraction mechanism.

## CONCLUSION

Moderate hypothermia (28º-29ºC) during heart surgery with extracorporeal circulation has led to a decrease in the amplitude of distortion product otoacoustic emissions in the cohort of patients analyzed.

## References

[1] Fetterman BL (2001). Distortion-product otoacoustic emissions and cochlear microphonics: Relationships in patients with and without endolymphatic hydrops. Laryngoscope.

[2] Lonsbury-Martin BL, Martin GK (1990). The clinical utility of distortion-product otoacoustic emissions. Ear Hear.

[3] Perlman HB, Kimura R, Fernandez C (1959). Experiments on the temporary obstruction of the internal auditory artery. Laryngoscope.

[4] Ness J, Stankiewitz J, Kaniff T (1993). Sensorineural hearing loss associated with aortocoronary bypass surgery: a prospective analysis. Laryngoscope.

[5] Shapiro MJ, Purn JM, Raskin C (1981). A study of the effects of cardiopulmonary bypass surgery on auditory function. Laryngoscope.

[6] Coats AC (1965). Temperature effects on the peripheral auditory apparatus. Science.

[7] Van Dijk P, Wit HP, Segenhout JM (1989). Spontaneous otoacoustic emissions in the European edible frog (Rana esculenta) spectral details and temperature dependence. Hear Res.

[8] Manley GA, Gallo L, Koppl C (1996). Spontaneous otoacoustic emissions in two gecko species, Gekko gecko and Eublepharis macularis. J Acoust Soc Am.

[9] Taschenberger G, Manley GA (1997). Spontaneous otoacoustic emissions in barn owl. Hear Res.

[10] Khovoles R, Freeman S, Sohmer H (1998). Effect of temperature on the transient evoked and distortion product otoacoustic emissions in rats. Audiol Neurootol.

[11] Seifert E, Lamprecht-Dinnesen A, Asfour B (1998). The influence of body temperature on transient evoked otoacoustic emissions. Br J Audiol.

[12] Seifert E, Brand K, Van Defleirdt K (2001). The influence of hypothermia on outer hair cells of the cochlea and its efferents. Br J Audiol.

[13] Musiek FE, Baran J (1997). Distortion product otoacoustic emissions: hit and false-positive rates in normal-hearing and hearing-impaired subjects. Am J Otol.

[14] Kemp DT (1978). Stimulated acoustic emissions from within the human auditory system. J Acoust Soc Am.

[15] Brownell WE (1990). Outer hair cell electromotility and otoacoustic emissions. Ear Hear.

[16] Veuillet E, Collet L, Duclaux R (1991). Effect of contralateral acoustic stimulation on active cochlear micromechanical properties in human subjects: dependence on stimulus variables. J Neurophysiol.

[17] Ashmore JF (1987). A fast motile response in guinea-pig outer hair cells: The cellular basis of the cochlear amplifier. J Physiol.

[18] Kalinec F, Holley MC, Iwasa KH (1992). A membrane-based force generation mechanism in auditory sensory cells. Proc Natl Acad Sci.

[19] Lonsburry-Martin BL, Whithead ML, Martin GK (1991). Clinical applications of otoacoustic emissions. J Speech Hear Res.

[20] Probst R, Harris FP, Alford BR, Jerger J, Jenkins HA (1997). Electrophysiologic Evaluation in Otolaryngology.

[21] Beattie RC, Jones RL (1998). Effects of relative levels of the primary tones on distortion product otoacoustic emissions in normal-hearing subjects. Audiol.

[22] Doyle KJ, Mc Laren CE, Shanks JE (2001). Effects of difluoromethylornithine chemoprevention on audiometry thresholds and otoacoustic emissions. Arch Otolaryngol Head Neck Surg.

[23] Whitehead ML, Lonsbury-Martin BL, Martin GK (1992). Evidence for two discrete sources of 2f1-f2 distortion-product otoacoustic emission in rabbit: I. Differential dependence on stimulus parameter. J Acoust Soc Am.

[24] Berninger E, Gustafsson LL (2000). Changes in 2f1-f2 acoustic distortion products in humans during quinine-induced cochlear dysfunction. Acta Otolaryngol.

[25] Sockalingam R, Freeman S, Cherny L (2000). Effect of high-dose cisplatin on auditory brainstem responses and otoacoustic emissions in laboratory animals. Am J Otol.

[26] Barenäs ML, Holgers KM (2000). Ototoxic Interaction between noise and pheomelanin: distortion product otoacoustic emissions after acoustical trauma in chloroquine-treated red, black and albino guinea pigs. Audiol.

[27] Stavroulaki P, Nikolopoulos TP, Psarommatis I (2001). Hearing evaluation with distortion-product otoacoustic emissions in young patients undergoing hemodialysis. Clin Otolaryngol.

[28] Delgado RE, Ozdamar O, Rahman S (2000). Adaptive noise cancellation in a multimicrophone system for distortion product otoacoustic emission acquisition. IEEE Trans Biom Eng.

[29] Le Bourgeois III HW, Anand VK, Mc Auley JR (2000). Effect of tympanic perforations on the detection of distortion-product otoacoustic emissions. ENT-Ear Nose Throat J.

[30] Slogoff S, Girgs KZ, Keats AS (1982). Etiologic factors in neuropsychiatric complications associated with cardiopulmonary bypass. Anesth Analog.

[31] Breuer AC, Furlan AJ, Hanson MR (1983). Central nervous system complications of coronary artery bypass graft surgery: a prospective analysis of 421 patients. Stroke.

[32] Shaw PJ, Bates D, Cartlige NEF (1985). Early neurological complications of coronary artery bypass surgery. Br Med J.

[33] Ferry PC (1990). Neurologic sequelae of open heart surgery in children. Am J Dis Children.

[34] Lonsbury-Martin BL, Harris FP, Stagner BB (1990). Distortion product emissions in humans. I. Basicproperties in normally hearing subjects. Ann Otol Rhinol Laryngol.

[35] Aremberg IK, Allen GW, De Boer A (1972). Sudden deafness immediately following cardiopulmonary bypass. J Laryngol Otol.

[36] Meri S, Aronen M, Leijala M (1988). Complement activation during cardiopulmonary bypass in children. Complement.

[37] Anderson SD, Kemp DT (1979). The evoked cochlear mechanical response in laboratory primates. Arch Otorhinolaryngol.

[39] Doyle WJ, Fria TJ (1985). The effects of hypothermia on the latencies of the auditory brain-stem response (ABR) in the rhesus monkey. Electroenc Clin Neurophysiol.

[40] Hett DA, Smith DC, Pilkingston SN (1995). Effect of temperature and cardiopulmonary bypass on the auditory evoked response. Br J Anaesth.

[41] Rodriguez RA, Audenaert SM, Austim III EH (1995). Auditory evoked responses in children during cardiopulmonary bypass: report of cases. J Clin Neurophysiol.

[42] Greeley WJ, Hern FH, Ungerleider RM (1991). The effect of hypothermic cardiopulmonary bypass and total arrest on cerebral metabolism in neonates, infants and children. J Thorac Cardiovasc Surg.

[44] Ohlemiller KK, Siegel JH (1992). The effects of moderate cooling on gross cochlear potentials in the gerbil: basal and apical differences. Hear Res.

